# Translation and cross-cultural adaptation of the Japanese version of the Assessment of Interprofessional Team Collaboration Scale-II (J-AITCS-II)

**DOI:** 10.15694/mep.2019.000195.1

**Published:** 2019-10-23

**Authors:** Yu Yamamoto, Junji Haruta

**Affiliations:** 1 University of Tsukuba

**Keywords:** Assessment tool, Interprofessional collaboration, Cultural adaptation, Exploratory factor analysis, Japan

## Abstract

This article was migrated. The article was marked as recommended.

**Introduction:** Several countries have developed interprofessional competency frameworks for interprofessional collaboration (IPC) that have the potential to improve healthcare processes and outcomes. However, there is no consensus on the assessment of IPC in clinical practice. The Assessment of Interprofessional Team Collaboration Scale-II (AITCS-II) is a validated questionnaire used to measure IPC among team members.

**Methods:** We translated the original AITCS into Japanese according to the Guidelines for the Process of Cross-Cultural Adaptation of Self-Report Measures and verified its reliability and cultural adaptability. Healthcare staff at a hospital and welfare facilities with multiple departments were asked to complete the Japanese version of the AITCS.

**Results:** The data of 558 responses were analyzed. Factor analysis of the Japanese version of the AITCS-II identified two factors: “Patient-centered collaborative care” and “Teamwork among healthcare professionals” with a Cronbach’s α > 0.7 for all items and subscales.

**Discussion:** The identified factors differed from those in the original questionnaire, which may be warranted considering the differences in the relationship between patients and healthcare professionals in Japan compared to Western countries. The Japanese version of the AITCS-II (J-AITCS-II) is a reliable and valid questionnaire, and further research should confirm its robustness and usefulness for improving IPC.

## Introduction

In recent years, economic and social factors have produced gaps in health conditions, and even in wealthy countries, these gaps are spreading. For example, social issues such as susceptibility to disease or risk of developing a severe condition due to poverty, and physical and psychosocial problems such as abuse and violence related to lack of social support ( World Health Organization, 2003) are increasing. Such complicated relationships between social determinant factors and disease are making it difficult to provide healthcare services. Further, the development of specializations in individual health care fields have made it more difficult to provide integrated services ( World Health Organization, 2010). To overcome such problems, professionals in different specialized fields must cooperate to provide healthcare services according to individual patients’ and service users’ needs. WHO refers to interprofessional collaboration (IPC) as follows: “Collaborative practice in health-care occurs when multiple health workers from different professional backgrounds provide comprehensive services by working with patients, their families, and carers” ( World Health Organization, 2010). In Canada, IPC was defined as the process of developing and maintaining effective interprofessional working relationships with learners, practitioners, patients/clients/families and communities to enable optimal health outcomes. Elements of collaboration include respect, trust, shared decision making, and partnerships( Canadian Interprofessional Health Collaborative, 2010). To ensure smooth IPC, medical professionals must be provided with opportunities to mutually learn from each other and from people in different specialized fields ( World Health Organization, 2010).

According to the Centre for the Advancement of Interprofessional Education (CAIPE), interprofessional education (IPE) arises when two or more professions learn about, from and with each other to enable effective collaboration and improvement of health outcomes ( CAIPE, 2002). To practice IPC and IPE, the skills required by healthcare professionals need to be determined and the outcomes based on these skills need to be evaluated. Such a practice is termed “competency-based education” and is the current trend in medical education. Competency frameworks required for the collaboration of healthcare professionals have been developed in the United States, the UK, Canada and Australia ( Thistlethwaite *et al.*, 2014). In Japan, the “Interprofessional Competency Framework” reflecting Japanese culture was developed in 2016 following the opinions of healthcare professionals and groups ( Haruta *et al.*, 2018).

Within the Japanese healthcare setting is embedded a culture in which doctors and nurses have significant power over other professionals, and the relationship between professionals can change depending on the members present at a particular time. For example, healthcare professionals other than doctors and nurses tend to avoid leadership roles. Furthermore, given that Japanese culture is a high context culture, professionals feel that they understand each other even when insufficient detail is given. Considering such cultural characteristics, the conceptual difference between the terms “team” and “collaboration” may not be obvious in Japan ( Haruta *et al.*, 2018). Therefore, taking such cultural backgrounds into consideration for common competency frameworks for healthcare professionals, from students, teachers, and practitioners to administrators, might be useful for systematic development of IPE in the future. However, there is no consensus on how to evaluate collaboration performance, even in countries where competencies have already been developed ( Rogers *et al.*, 2017).

There are currently few evaluation tools for interprofessional collaboration to have been developed in Japan or that are translated into Japanese. The Chiba Interprofessional Competency Scale (CICS29) was uniquely developed by Chiba University to measure the competency of interprofessional collaborative practice ( Sakai *et al.*, 2017). The Readiness for Interprofessional Learning Scale (RIPLS) quantitatively measures the readiness of students and professionals for IPE ( Tamura *et al.*, 2012; Oishi *et al.*, 2017), and the Interprofessional Facilitation Scale (IPFS) assesses educators’ skills in facilitating IPE ( Haruta, Breugelmans and Nishigori, 2018). However, no assessment tool has been developed regarding collaboration practices in clinical settings. The Assessment of Interprofessional Team Collaboration Scale (AITCS) is a measurement tool developed in Canada for evaluating collaboration within teams across various practice settings and the integration of patient involvement as part of team practice ( Orchard *et al.*, 2012). AITCS has been validated for its internal consistency, reliability and construct validity. In its original form, it consists of 37 items and 3 subscales representing discrete elements of interprofessional care, including: (1) Partnership/shared decision making, (2) Cooperation and (3) Coordination. In 2018, the shortened version of AITCS (AITCS-II) was developed and its validity and reliability were confirmed ( Orchard *et al.*, 2018). AITCS-II consists of the same 3 subscales as the original version. There are several potential applications for the AITCS-II in research, including continuing education, performance assessment, and evaluating team practice. AITCS has been translated into German, Spanish, Portuguese, and Rwandan French ( Orchard *et al.*, 2018). However, it is unclear whether AITCS-II can be applied in Japan, considering that Japanese attitudes differ from those in Western countries. The Japanese versions of RIPLS and IPFS differ from the original scales possibly to reflect Japanese education and cultural background in the questionnaires’ factor structures.

Examining the cultural adaptability of AITCS-II may reveal that AITCS-II is applicable in Asian countries that are culturally similar to Japan. Furthermore, this evaluation may improve the quality of collaborations. Therefore, the purpose of this research was to develop a Japanese version of the AITCS-II (J-AITCS-II), and to examine its validity and cultural adaptability.

## Methods

### Translation and cross-cultural adaptation

**Figure 1.**
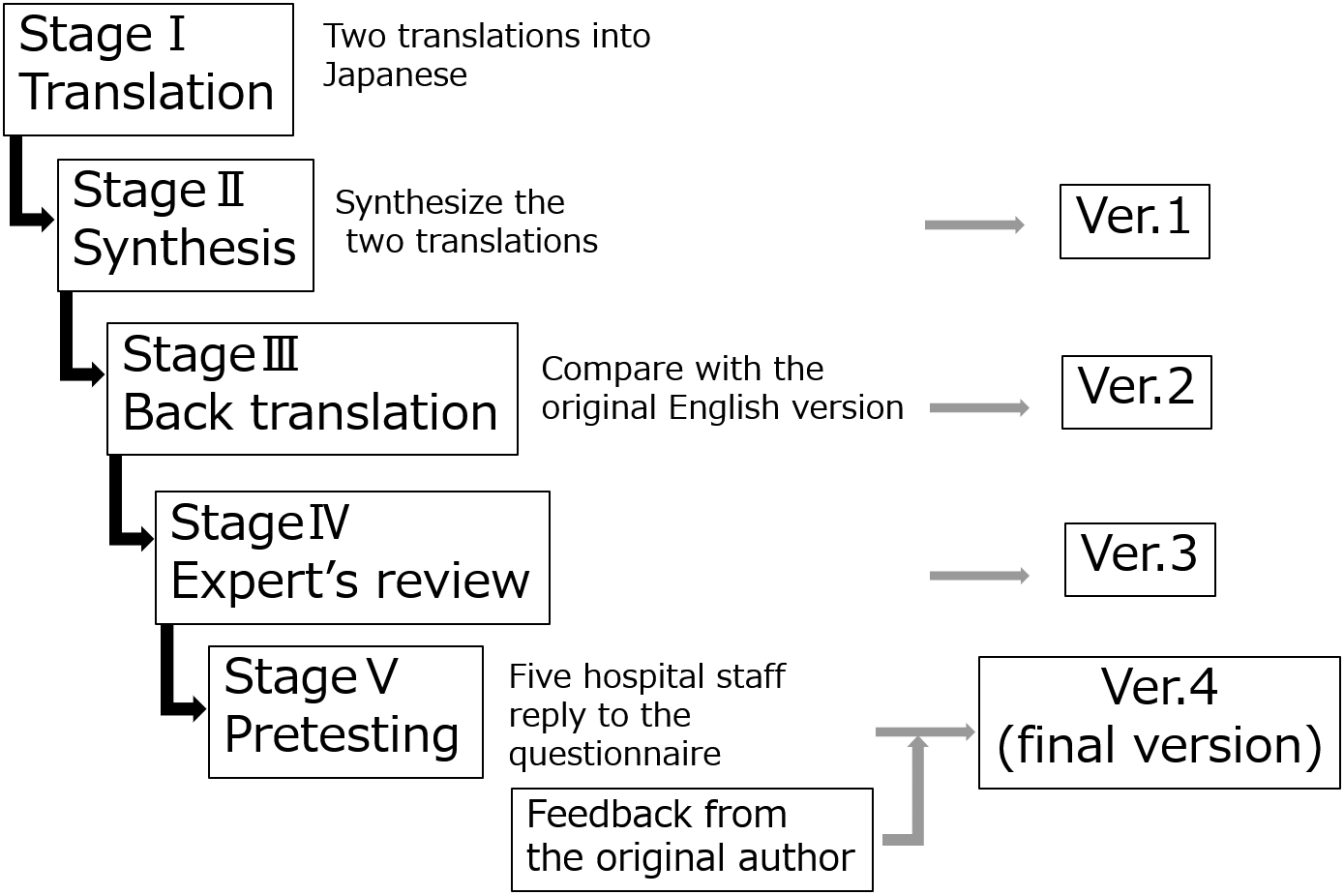
The stages of cross-cultural adaptation

Modified from Beaton *et al.*, 2000.

With consent from the original author, we translated the AITCS into Japanese based on the Guidelines for the Process of Cross-Cultural Adaptation of Self-Report Measures ( Beaton *et al.*, 2000) to reach equivalence between the original and Japanese versions of the AITCS ( Figure 1). Two translators, the first author (YY) and second author (JH), independently translated the AITCS into Japanese (Stage I Translation). The translations were compared and differences in interpretation and expression were discussed, and a new Japanese version was created (Ver.1) (Stage II Synthesis). We asked two people with native English proficiency and Japanese competency who were not involved in this study and had no knowledge of the original English version of the AITCS to translate Ver.1 back to English (Ver.BT).

We compared the original English version with Ver.BT, and by examining differences in expression, we revised Ver.1 (Ver.2) (Stage III Back translation). We subsequently asked an expert in IPE to examine the validity of Ver.2, and we revised Ver.2 based on this feedback (Ver.3) (Stage IV Expert review). We distributed Ver.3 to five hospital staff (a nurse, pharmacist, speech therapist, occupational therapist, and registered dietitian) and asked them to provide responses and to comment on the questionnaire. With these comments and the original author’s feedback on Ver.BT, we further modified the expression and created the final version (Ver.4) (Stage V Pretesting)

### Date collection

In July 2017, we distributed the Japanese version of the AITCS to all departments of X hospital and welfare facilities. X hospital is a general hospital located in the Chubu region. It has 360 beds, of which 324 are for acute care and 36 are for long-term care. There are 32 clinical departments in the hospital, and the associated welfare facilities include a visiting nursing station, dialysis clinic, and nursing home. The hospital conducts acute care as well as chronic care. The number of staff was 709 as of March 1, 2017, including 506 nurses, 99 doctors and dentists, 33 clinical technologists, 23 rehabilitation therapists, 16 pharmacists, and 14 clinical engineers.

### Ethical considerations

All respondents provided written consent to participate in this study. This research has been approved by the Ethics Committee of the University of Tsukuba (approval number: 1066).

### Statistical analysis

A series of psychometric analyses were performed on the Japanese version of the AITCS. Since AITCS-II was initially developed while conducting this research with the aim of producing a shorter and more sophisticated questionnaire, we decided to extract and analyze 23 items of AITCS-II from 37 items of AITCS. First, we excluded data with missing values. Second, exploratory factor analysis was conducted to explore the structure of the questionnaire items and these factors were compared to those of the original English version of AITCS-II. In factor analysis, the promax rotation was adopted in conjunction with the maximum likelihood method. Factor selection was based on Kaiser’s eigen values >1.0. In the process, we estimated the number of factor loadings. The cutoff value of factor loadings that suggests involvement in each factor was set to 0.4. Cronbach’s α was calculated to measure internal consistency. We also checked the histogram for each item and confirmed the existence of a ceiling effect and floor effect.

All data were analyzed using IBM SPSS 25 (IBM Japan, Tokyo, Japan).

## Results/Analysis

### Translation and cross-cultural adaptation

In the process of preparing Ver.1 to Ver.4, 14 items out of 37 were revised by reflecting the back translation, pretesting, expert opinion, and feedback from the original author. In the process of translating the questionnaire into Japanese, every effort was made to modify unnatural expressions into more natural expressions without changing the meaning of the original version ( Table 1). In Stage I Translation, and Stage II Synthesis, there were some discrepancies with the translators’ translating English into Japanese concerning differences in selected words and the structure of sentences, and expressions difficult to translate directly in Japanese. For example, in item 12 the expression “process of care ..” in the original version was translated in different ways by translators. We discussed that the use of simply “care..” would facilitate understanding of the item in Japanese. In addition, since Japanese healthcare systems and culture differ from those of Canada, where original AITCS was developed, we selected and revised item expressions on the basis of whether Japanese healthcare professionals in clinical settings could understand each item or not. For example, since “social service” in item 22 is an unclear expression in Japanese, we changed it to “welfare” in the Japanese version. In the Stage III Back translation, there was a Japanese expression in item 10 which was unclear when compared with the original item. For this item, we determined that “patients participating” would better clarify the meaning of “include patients” in the Japanese context.

**Table 1.** Changes made after cross-cultural adaptation

Item no.	Original AITCS (English)	Translation Ver.1(Japanese)	Direct translation of translation ver.1 (English)	Back translation (English)	Finaltranslation (Japanese)	Direct translation of Final translation (English)
**Changes made after the back translation**						
10	**Include patients** in setting goals for their care	...kanja mo fukumete okonau	involve patients	Involve patients when setting care goals	...mo sanka siteiru	**patients participating**
**Changes made after expert opinions**						
15	understand **the boundaries** of what each other can do	...tagai ga dekiru koto no kyoukai wo	the boundaries of what each other can do	understand the limits of what each other can do	...tagai ga dekiru koto no hani wo	Understand **the scope** of what each other can do
**Changes made after the feedback from author**						
2	Share **the power** with each other	...syokusyu ga motsu nouryoku wo...	the ability that professionals have	Share professional skills with each other	...syokusyu ga motsu kengen wo...	**the authority** that professionals have
5	Are open and honest with each other	Tagai ni socchoku de seijitsu dearu	We are frank and sincere with each other	Are frank and honest with each other	Tagai ni tasya no kangae wo yoku kiki seijitsu dearu	We listen to others’ opinion carefully and are sincere

### Respondent characteristics

A total of 689 participants responded to the AITCS. After excluding data with missing values, 558 responses were used for analysis. Among the participants, 26.0% were male (n = 145), 71.7% were female (n = 400) and 2.3% (n = 13) did not provide their sex. The staff members consisted of a variety of professionals, including nurses (n = 240), doctors (n = 46), administration clerks (n = 39), nursing care workers (n = 37), physical therapists (n = 28), medical clerks (n = 26), pharmacists (n = 19), nursing assistants (n = 16), occupational therapists (n = 14), obstetric nurses (n = 14), medical technologists (n = 13), clinical engineering technologists (n = 10), registered dietitians (n = 9), care managers (n = 7), certified social workers (n = 6), speech therapists (n = 5), radiological technologists (n = 5), caregivers (n =4), dental hygienists (n = 4), orthoptists (n = 2), one assistant, one safety manager, one health nurse, one pharmacy assistant, one clinical psychotherapist, and nine other occupations. The average experience in a clinical setting was 12.2 years and the average years worked in the current department was 5.3 years ( Table 2).

**Table 2.** Respondents’ characteristics

Gender, N(%)		Female 400 (71.7) No response (2.3)
Average experience in a clinical setting(years, mean±SD)		12.2±9.8
Average time worked in the current department(years, mean±SD)		5.3±6.1
Profession		
	Nurse	240
	Doctor	46
	Administration clerk	39
	Nursing care worker	37
	Physical therapist	28
	Medical clerk	26
	Pharmacist	19
	Nursing assistant	16
	Occupational therapist	14
	Obstetric nurse	14
	Medical technologist	13
	Clinical engineering technologist	10
	Registered dietitian	9
	Care manager	7
	Certified social worker	6
	Speech therapist	5
	Radiological technologist	5
	Caregiver	4
	Dental hygienist	4
	Orthoptist	2
	Assistant, Safety manager,Health nurse, Pharmacy assistant, Clinical psychotherapist	1
	Other occupations	9

### Exploratory factor analysis

The Kaiser-Meyer-Olkin (KMO) value was 0.961, which is greater than the suggested minimum value of 0.6 ( Kaiser, 1974; Cerny and Kaiser, 1977), and Bartlett’s test for sphericity was significant (p＜0.001, χ2=10491.747, df =253). Factor analysis showed that the cumulative contribution rate for the first two factors was 64.5%, while the individual contribution rates were 53.8% for the first factor and 10.7% for the second. Factor loadings after rotation are shown in Table 3.

Finally, factor analysis identified 14 items for factor 1 and 9 items for factor 2. Cronbach’s α value was >0.7 for each of these factors. We obtained a Cronbach’s α = 0.96 for all items, and α = 0.95 for the subscale of factor 1 and α = 0.92 for that of factor 2. On checking the histograms for each item, we were unable to confirm a ceiling or floor effect.

**Table 3.** Results of factor analysis

	Items(as in original AITCS-II version)		Factor1	Factor2
Factor 1 Patient-centered collaborative care	24	use consistent communication with to discuss patient care	**0.914**	-0.062
	25	are involved in goal setting for each patient	**0.911**	-0.047
	35	encourage each other and patients and their families to use the knowledge and skills that each of us can bring in developing plans of care	**0.886**	-0.032
	37	work with the patient and his/her relatives in adjusting care plans	**0.882**	-0.015
	20	meet and discuss patient care on a regular basis	**0.821**	0.009
	27	support the leader for the team varying depending on the needs of our patients	**0.807**	0.007
	22	coordinate health and social services (e.g. financial, occupation, housing, connections with community, spiritual) based upon patient care needs	**0.805**	-0.048
	29	openly support inclusion of the patient in our team meetings	**0.753**	-0.097
	13	encourage and support open communication, including the patients and their relatives during team meetings	**0.739**	0.056
	28	together select the leader for our team	**0.659**	0.000
	12	listen to the wishes of their patients when determining the process of care chosen by the team	**0.643**	0.171
	10	include patients in setting goals for their care	**0.642**	0.117
	16	understand that there are shared knowledge and skills between health providers on the team	**0.481**	0.314
	14	use an agreed upon process to resolve conflicts	**0.448**	0.369
Factor 2 Teamwork in healthcare professionals	4	respect and trust each other	-0.205	**0.993**
	5	are open and honest with each other	-0.154	**0.957**
	19	establish a sense of trust among the team members	-0.032	**0.785**
	2	share power with each other	0.067	**0.758**
	9	strive to achieve mutually satisfying resolution for differences of opinions	0.073	**0.728**
	15	understand the boundaries of what each other can do	0.062	**0.690**
	6	make changes to their team functioning based on reflective reviews	0.172	**0.562**
	1	apply a unique definition of Interprofessional collaborative practice to the practice setting	0.272	**0.519**
	11	equally divide agreed upon goals amongst the team	0.367	**0.429**
Eigenvalue			12.371	2.471
Percent variance explained			53.787	10.744
Cronbach’s α			0.952	0.923

## Discussion

Using a cross-cultural adaptation process, we translated and validated the 23 items of the original AITCS-II to create the J-AITCS-II questionnaire. The content validity, reliability and internal consistency was maintained in the J-AITCS-II.

Exploratory factor analysis demonstrated that all items in the J-AITCS-II had good internal consistency, and two factors were extracted. The original AITCS-II consists of three factors, named “Partnership”, “Cooperation”, and “Coordination”. After comparing J-AITCS-II with the original version, we labeled factor 1 “Patient-centered collaborative care” and factor 2 “Teamwork among healthcare professionals”.

In the original version, the authors first fixed each factor before developing the items to fit the factors. In contrast, we applied factor analysis to each item, and identified a new factor structure. Despite the fact that more than half of the items of factor 1 were classified as “Partnership” in the original AITCS-II, we did not name this factor “Partnership” but called it “Patient-centered collaborative care” after considering the meaning of all of the items comprising factor 1. “Partnership” is a relationship that respects the roles and contributions of each participant ( Orchard *et al.*, 2012), and voluntary participation of patients and proper sharing of information are necessary. However, particularly in the case of elderly people, Japanese patients tend to leave important decisions to family members rather than making their own decisions ( Masaki, Ishimoto and Asai, 2014). Japanese medical staff also tend to prioritize the opinions of the patients’ family ( Ruhnke *et al.*, 2000). Furthermore, in Japan, the history of patient participation in clinical settings such as informed consent is relatively short ( Akabayashi and Slingsby, 2006). Therefore, many healthcare professionals may feel uncomfortable conveying extenuating circumstances directly to patients or involving patients in team decision-making processes. For example, there are some cases, such as in cancer or severe disease, in which healthcare professionals do not share information on the disease name or prognosis with the patient but only with the patient’s family ( Hattori *et al.*, 1991; Powell, 2006). Japanese physicians prioritize not exacerbating patients’ anxiety over telling them the facts. Based on this background, we considered it reasonable to conclude that a factor like “Partnership” may not be rooted in the Japanese medical setting. Instead, we determined that “Patient-centered collaborative care” was a more suitable name for factor 1. Although we have described the current situation in Japan, similar cases are recognized in other countries, especially with regard to decision-making. In Asian and European countries or in America, many people desire more autonomous decision-making ( Gravel, Légaré and Graham, 2006; Deber *et al.*, 2007; Ambigapathy, Chia and Ng, 2016), but patient participation in decision-making is still uncommon.

The factors “Patient-centered collaborative care” and “Teamwork among healthcare professionals” comprised items that were clearly divided in terms of whether or not they were associated with “patients”. This trend is not recognized in the original version. Given that the AITCS was originally developed for the evaluation of patients’ participation in their care process, it may appear strange that items which are associated with “patient” gather in one group. To understand this, some cultural background behind the Japanese terms “Uchi” and “Soto” is necessary. “Uchi” refers the in-group such as those within the same organization or community, and “Soto” refers to the out-group, or those other than “Uchi”. Members of “Uchi” have strong connections with each other and tend to exclude the people of “Soto” ( Nakane, 1967). Japanese healthcare professionals harbor this conceptual belief and tend to consider themselves members of “Uchi” and patients as “Soto” ( Miyake, 1994). Therefore, when Japanese healthcare professionals imagine the term “team”, they tend to picture a team of only healthcare professionals. In addition, rather than considering patients as members of the team, they consider them participants of the team only when necessary. This is one reason why we named factor 2 “Teamwork among healthcare professionals” and not simply “cooperation”. A similar culture exists in Asian countries, such as Korea and China, suggesting that this discussion can be applied to other countries ( Osaki, 2008).

Summarizing the reliability and validity of this questionnaire, as described so far, Cronbach’s α value for each item and each factor showed good internal consistency. Exploratory factor analysis was performed to confirm construct validity and the reasons why a new factor structure was found were discussed. Finally, the authors of the original article checked the ver. BT and all feedback was considered. Through these procedures, we considered that the J-AITCS-II retained both item-level and scale-level reliability and validity.

Our study has several limitations. First, the original meaning of some of the items may have been changed in the process of translation. However, we expect that this effect was small because we conducted two back translations as recommended by the guidelines for linguistic validation of translations ( Beaton *et al.*, 2000), and the translation process was sufficiently robust to clarify vague meanings. Furthermore, the expressions of AITCS-II are slightly different from those of the original AITCS. We used the translation of the original AITCS for each item and found no significant change in meaning, indicating that there is no significant problem in using it. Second, this study was conducted at a single facility; therefore, our findings may not accurately reflect the current general situation in Japanese healthcare settings. However, X hospital comprises a variety of healthcare professionals, from whom we received a response rate of 97% (689/709), which is higher than that of a well-known study by Cook et al. ( Cook, Dickinson and Eccles, 2009), suggesting that our participants may be sufficiently diverse. Third, the rate of missing values in this study was 19%. This appears large, and might have warranted imputation of data. We referred to some studies that performed exploratory factor analysis without imputation despite a certain number of missing values. ( Tamura *et al.*, 2012) It is assumed that the rate of missing values does not have a big influence on the factor structure because this study has a relatively large number. We will consider imputation of missing values in confirmatory factor analysis in subsequent research. Fourth, it is possible that the reliability and validity of this questionnaire required more assessment than that outlined in the guideline we used for this study. To achieve this, further testing, such as correlations with similar questionnaires and comparisons between groups known to differ in their background, should be considered.

Further research is required to confirm that the J-AITCS-II is a robust assessment tool and that it enables the effective evaluation of collaborative practice in Japanese healthcare settings. In the future, it may be possible to apply the AITCS-II to the Confucian Asian cluster, of which Japan is a member. In addition, this unique Japanese assessment tool will make it possible to compare the characteristics of IPE/IPW in Japan with those of other countries. We expect that such studies will improve the practice of interprofessional collaboration and IPE.

## Conclusion

We translated the AITCS-II into Japanese using a reliable procedure and verified its content validity, reliability and internal consistency. Two factors were extracted in the exploratory factor analysis, and the grouping of items differed from that in the original questionnaire. The Japanese custom of distinguishing healthcare professionals from patients may have warranted the difference in grouping of factors. Further research to confirm the robustness of the J-AITCS-II is warranted.

## Take Home Messages

• J-AITCS-II was developed and its content validity, reliability and internal consistency were confirmed.
• J-AITCS-II has several potential applications in evaluating team practice, continuing education and research into IPE/IPW in Japan.
• J-AITCS-II can be applied to the Confucian Asian cluster to improve the practice of interprofessional collaboration and IPE.
• The use of J-AITCS-II has made it possible to compare the actual situation of Japanese IPE/IPW with that in other countries around the world.

## Notes On Contributors

Yu Yamamoto, MD, is an Assistant Professor, Department of Family Medicine, General Practice and Community Health, Faculty of Medicine, University of Tsukuba, Japan.

Junji Haruta, MD, PhD, is an Associate Professor, Department of Primary Care and Medical Education, Faculty of Medicine, University of Tsukuba, Japan.

## Declarations

The author has declared that there are no conflicts of interest.

## Ethics Statement

This research has been approved by the Ethics Committee of the University of Tsukuba (approval number: 1066).

## External Funding

This research has been done as part of a larger project funded by the Grant-in-Aid for Young Scientists(B) of Grant Number 16k19170.
