# Supplementary material for: Translation and cross-cultural adaptation of the Japanese version of the Assessment of Interprofessional Team Collaboration Scale-II (J-AITCS-II)
Source: MedEdPublish (2016). 2019 Oct 23;8:195. [Version 1] doi: 10.15694/mep.2019.000195.1 (PMC10712457; doi:10.15694/mep.2019.000195.1)
Supplement: Peer review history

# Reviewer report: Reviewer response for version 1

**DOI:** 10.21956/mep.19933.r30238

**Published:** 2020-05-12

**Authors:** Richard Hays

**Affiliations:** 1 James Cook University

This review has been migrated. The reviewer awarded 4 stars out of 5 Translation of an international tool into another language and culture is a complex process. I am not familiar with the particular tool, but accept that it may be appropriate for a range of IPE contexts, with some adaptation. I think the authors should be congratulated for their approach, which appears comprehensive. Some changes in wording are to be expected, as languages sometimes encapsulate different conceptual meaning in words and phrases. This paper is therefore really a report of a validation, particularly of the language translation. It would be good now to see the tool applied in other Japanese (and perhaps other Asian nations) health facilities, to see if the tool detects meaningful differences between them. That would soidify the next step - cultural validation - which I am not sure can be done in only one hospital system. Discussion of that issue would have earned a 5th star (perhaps leading to another project) but the paper should be of interest to all involved in IPE.

Reviewer Expertise:

NA

No decision status is available

# Reviewer report: Reviewer response for version 1

**DOI:** 10.21956/mep.19933.r30240

**Published:** 2020-02-04

**Authors:** Ken Masters

**Affiliations:** 1 Sultan Qaboos University

This review has been migrated. The reviewer awarded 5 stars out of 5 An interesting paper dealing with the translation and cross-cultural adaptation of the Japanese version of the Assessment of Interprofessional Team Collaboration Scale-II (JAITCS-II). Accurate translation is never easy, and is made more complex when one wishes to ensure that the professional spirit of a document (beyond the words) is accurately translated, while simultaneously taking into account cultural and social differences.The authors have done well to lay out the needs and current literature on the topic. The process of back-translation by those not familiar with the original, and then comparison and verification was an important step in ensuring documentation veracity and authenticity. Further commenting by other health professionals increased these.The validity of the tool was further strengthened by its use and Cronbach’s alpha testing.Although, as the author’s state, further issues may emerge when the tool is used elsewhere, they are to be congratulated on having followed strict procedures in their construction of this tool for IPC in Japan. I look forward to seeing future research papers in which use of the tool becomes standard.

Reviewer Expertise:

NA

No decision status is available

# Reviewer report: Reviewer response for version 1

**DOI:** 10.21956/mep.19933.r30239

**Published:** 2019-10-24

**Authors:** Felix Silwimba

**Affiliations:** 1 University of Lusaka

This review has been migrated. The reviewer awarded 5 stars out of 5 this i a well articulated study. its worth considering and replicating in low and middle income countries healthcare delivery.

Reviewer Expertise:

NA

No decision status is available
